# Comprehensive metrological and content analysis of the income inequality research in health field: A bibliometric analysis

**DOI:** 10.3389/fpubh.2022.901112

**Published:** 2022-09-14

**Authors:** Guocheng Xiang, Jingjing Liu, Shihu Zhong, Mingjun Deng

**Affiliations:** ^1^School of Business, Hunan University of Science and Technology, Xiangtan, China; ^2^College of Economics and Trade, Hunan University of Technology and Business, Changsha, China; ^3^Department of Applied Economics, Shanghai National Accounting Institute, Shanghai, China; ^4^Research Center of Big Data and Intelligent Decision, Hunan University of Science and Technology, Xiangtan, China

**Keywords:** income inequality, health, bibliometric analysis, CiteSpace, knowledge mapping

## Abstract

The association between income inequality in a society and the poor health status of its people has attracted the attention of researchers from multiple disciplines. Based on the ISI Web of Science database, bibliometric methods were used to analyze 546 articles related to income inequality research in health field published between 1997 and 2021. We found that the USA contributed most articles, the Harvard Univ was the most influential institution, Social Science & Medicine was the most influential journal, and Kawachi I was the most influential author; the main hotspots included the income inequality, income, health inequality, mortality, socioeconomic factors, concentration index, social capital, self-rated health, income distribution, infant mortality, and population health in 1997–2021; the cardiovascular disease risk factor, social capital income inequality, individual mortality risk, income-related inequalities, understanding income inequalities, income inequality household income, and state income inequality had been the hot research topics in 1997–2003; the self-assessed health, achieving equity, income-related inequalities, oral health, mental health, European panel, occupational class, and cardiovascular diseases had been the hot research topics in 2004–2011; the adolescent emotional problem, South Africa, avoidable mortality, rising inequalities, results from world health survey, working-age adult, spatial aggregation change, prospective study, and mental health-empirical evidence had been the hot research topics in 2012–2021; there were 11 articles with strong transformation potential during 2012–2021. The research results of this paper are helpful to the scientific understanding of the current status of income inequality research in health field.

## Introduction

Wide income inequality1 in a society has been associated with worse aggregate health (1). Equal societies are healthier as they have higher social cohesion, good social relation, and less stress (2). On the contrary, people living in unequal societies suffer from poor health in general. Income poverty, along with inequality, increases the risk of premature mortality and increased morbidity (3). The association between income inequality in a society and the poor health status of its people has attracted the attention of researchers from multiple disciplines (4). Who are the famous scholars in the income inequality research in health field? Which countries and institutions have close exchanges? What are the research subjects and development trends? These problems need further analysis. To solve this problem, it is necessary to use scientometric software (5), because bibliometric studies can provide new perspectives about the knowledge status and trends in a given field (6–8). However, to the best of our knowledge, only Wagstaff and van Doorslaer (9), Macinko et al. (10), and Pickett and Wilkinson (11) reviewed the large and growing body of literature on the relationship between income inequality and health outcomes, no bibliometric analysis on the income inequality research in health field at a global scale. Therefore, we aimed to visually demonstrate knowledge structures and developments within the income inequality research in health field using the bibliometric analysis method, to guide scholars and practitioners to determine the research interests and emerging themes of the income inequality research in health field, so as to enhance their understanding and evaluation of the income inequality research in health field.

This paper is organized as follows. After this introduction, Section 2 provides the primary research materials and methods. Section 3 presents the research findings and analyses. Section 4 presents the research-related conclusion.

## Materials and methods

### Data acquisition

**Figure 1:**
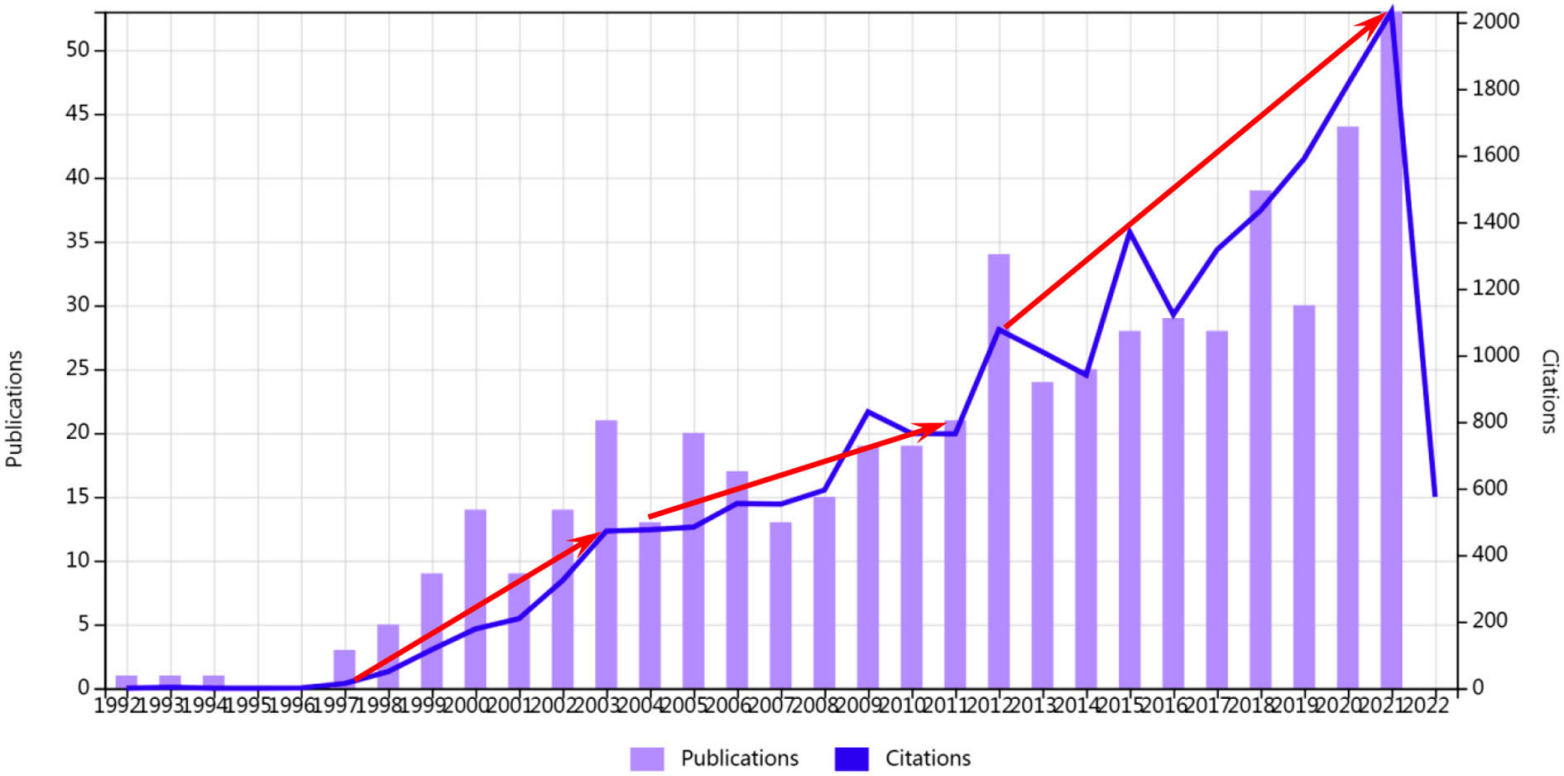
Times cited and publications over time.

This study regards the Web of Science (WOS) Core Collection as a data-collection platform according to data resources required in CiteSpace. The bibliometric search strategy can be described as the following: Title = (income inequality), Publication years = (1997-01-01 to 2021-12-31), Document types = (article), Indexes = (SSCI, SCI-EXPANDED, ESCI), Web of Science Categories = (Public Environmental Occupational Health or Health Care Sciences Services), Retrieval date: May 14, 2022. A total of 546 publications were selected. Regarding the time period of the retrieved data, we mainly considered the time range for the following reason: As shown in Figure 1, the number of papers published had been increasing since 1997.

### Bibliometric analysis

Bibliometrics is an interdisciplinary study that utilizes mathematics, statistics, and bibliography to quantitatively analyze academic literature (12). The common methods of bibliometrics include statistical analysis, citation analysis, and sharing analysis. In addition, mapping knowledge can show the relationship between the development process and structure of scientific knowledge, focus on the evolution process of a certain knowledge field, and help scholars understand the hot spots, frontiers, and trends of research in this field (13).

The statistical analysis (including journals, authors, countries, and institutions) can help the people who are interested in a field to grasp the basic information and development status of the literature quickly (14). We used the Online Analysis Platform for Bibliometrics to conduct visualization analysis of publication year, journal, and countries.

Partnership analysis mainly analyzes the relationship between countries, the relationship between institutions, and the relationship between authors. We used Online Analysis Platform for Bibliometrics (https://bibliometric.com/) and VOSviewer to analyze the cooperation network among countries, institutions, and authors.

The keyword co-occurrence analysis is an effective way to detect the hot topics and evolving research frontiers of particular research fields over time. The evolution of research topics has always been a research issue that researchers have been concerned about because it helps researchers to understand the development of certain areas, pioneer in that area, and finally determine corresponding research directions (15). In this paper, we made keyword co-occurrence network map by VOSviewer and monitored hot topics in the income inequality and health research through keyword co-occurrence network analysis.

Co-citation analysis makes it possible to map the intellectual structure of a research field, to detect trends in the research area (by the authors who work on these topics and the nexus between them), and to discover first-line studies and highlight high-impact discoveries (16). With the development of science and technology, many visualization tools have emerged in recent years, such as VOSviewer, CoPalRed, Bibexcel, Sci2, VantagePoint, CiteSpace, and Online Analysis Platform for Bibliometrics. CiteSpace is a strong tool to systematically learn about one field rapidly. The principle of CiteSpace is to label co-citation clusters and then use time-sliced snapshots to form timeliness and pivotal points (5). In this study, we used CiteSpace (Version 5.8.R3) to find major research areas in the knowledge domain and journal overlay maps. To better understand the development of the income inequality research in health field, we carried out co-citation analysis in three periods: (1) Set a time span from 1997 to 2003, set “years per slice = 1,” “node types = reference,” “Selection Criteria: g-index, k = 50,” and “Pruning = Pathfinder, and Pruning sliced networks” in CiteSpace to analyze their intellectual structure and the dynamics of co-citation clusters. (2) Set a time span from 2004 to 2011, set “years per slice = 1,” “node types = reference,” “Selection Criteria: g-index, k = 70,” and “Pruning = Pathfinder, and Pruning sliced networks” in CiteSpace to analyze their intellectual structure and the dynamics of co-citation clusters. (3) Set a time span from 2012 to 2021, set “years per slice = 1,” “node types = reference,” “Selection Criteria: g-index, k = 45,” and “Pruning = Pathfinder, and Pruning sliced networks” in CiteSpace to analyze their intellectual structure and the dynamics of co-citation clusters.

To realize co-citation analysis, we set a time span from 1997 to 2021 set “years per slice = 1,” “node types = reference,” and “Selection Criteria: g-index, k = 80” in CiteSpace to analyze their intellectual structure and the dynamics of co-citation clusters, respectively.

## Results and analyses

### Discipline co-occurrence analysis

**Figure 2:**
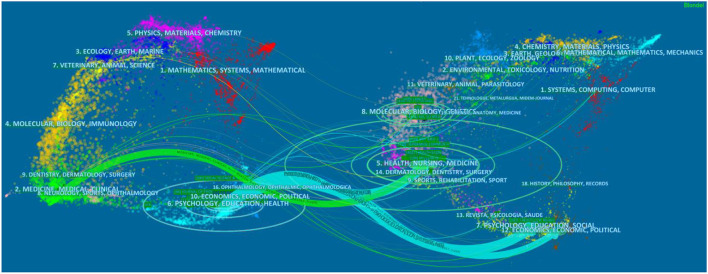
Dual-map overlay of literature on income inequality and health research.

*Via* the “dual-map overlay” function of CiteSpace, we are able to show the discipline co-occurrence network in Figure 2. The original map exhibits over 10,000 journals indexed in Web of Science, which are classified into different disciplines with different colors and located in different spots of both the source (or left) area and the reference (or right) area. For example, the discipline “ECONOMICS, ECONOMIC, POLITICAL” is in lake blue, and it ranks the 10th in the source area and the 12nd in the reference area (17). Then, we add the layer containing the 546 bibliographic records on the income inequality in “Public Environmental Occupational Health or Health Care Sciences Services” category, which turns into the colorful links between the source and the reference area.

From Figure 2, we see that the impact of the income inequality research in health field is very widespread from 1997 to 2021, but relatively more source journals are located in “2. MEDICINE, MEDICAL, CLINICAL,” “6. PSYCHOLOGY, EDUCATION, HEALTH,” and “9. DENTISTRY, DERMATOLOGY, SURGERY.” As for the distribution of the reference journals, “6. PSYCHOLOGY, EDUCATION, HEALTH” links going to “12. ECONOMICS, ECONOMIC, POLITICAL,” “7. PSYCHOLOGY, EDUCATION, SOCIAL,” and “5. HEALTH, NURSING, MEDICINE” account for higher percentage; “2. MEDICINE, MEDICAL, CLINICAL” links going to “5. HEALTH, NURSING, MEDICINE” account for higher percentage.

### Publication characteristics analysis

**Figure 3:**
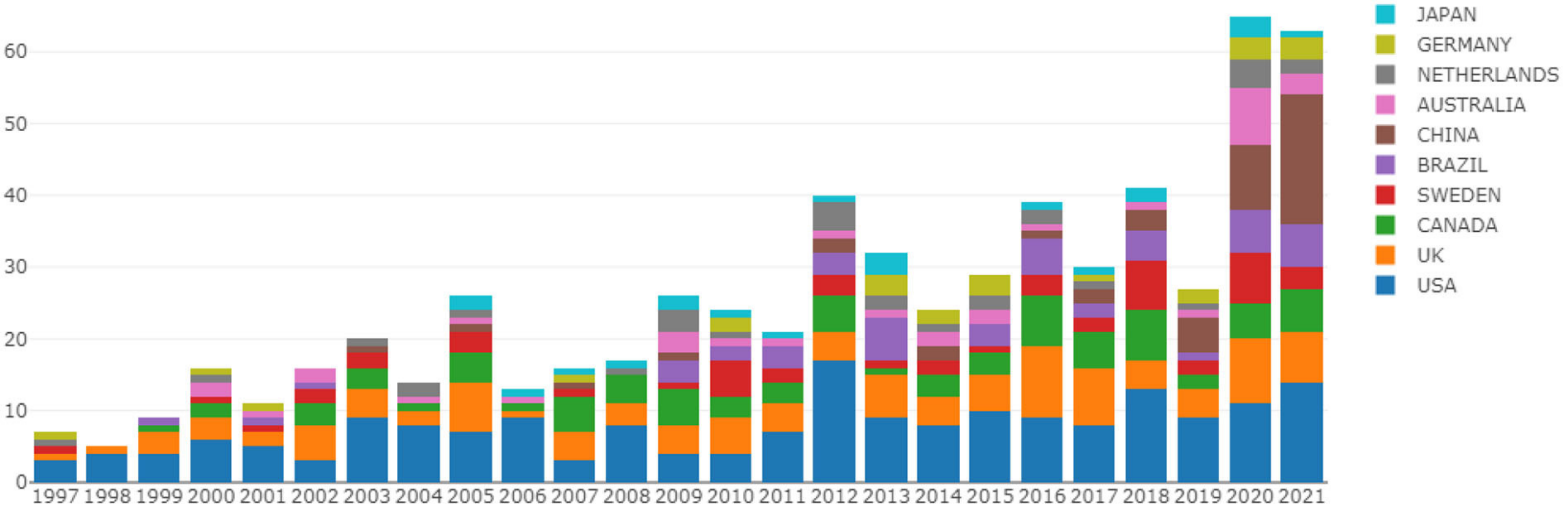
Number of papers published by countries each year.

The top 10 countries in the number of annual publications are shown in Figure 3 (Since the Online Analysis Platform for Bibliometrics can only automatically generate the top ten countries, the situation of annual publications in other countries cannot be seen in this figure). The number of articles published by scholars in the USA, UK, and CANADA has been relatively high every year from 1997 to 2021. The number of articles published by scholars in China had shown a rapid growth trend from 2019 to 2021.

As shown in Table 1 (1) among the top 10 publication titles in terms of publication volume, “Social Science Medicine,” “Journal of Epidemiology and Community Health,” and “International Journal for Equity in Health” published 100, 46, and 37 papers, respectively, from 1997 to 2021; (2) among the top 10 authors in the number of papers published, Kawachi I, Subramanian SV, and Barros AJD had published 37, 13, and 12 articles, respectively, from 1997 to 2021.

**Table 1:** Top 10 publication titles and authors.

**Publication titles**	**Record count**	**Authors**	**Record count**
Social Science & Medicine	100	Kawachi I	37
Journal of Epidemiology and Community Health	46	Subramanian SV	13
International Journal for Equity in Health	37	Barros AJD	12
BMC Public Health	23	Pabayo R	10
American Journal of Public Health	22	Gerdtham UG	9
International Journal of Environmental Research and Public Health	22	Gustafsson PE	9
Health Economics	15	Harper S	9
Journal of Health Economics	14	Mosquera PA	9
European Journal of Public Health	13	Van Doorslaer E	9
Health Place	13	Chiavegatto ADP	7

### Partnership analysis

**Figure 4:**
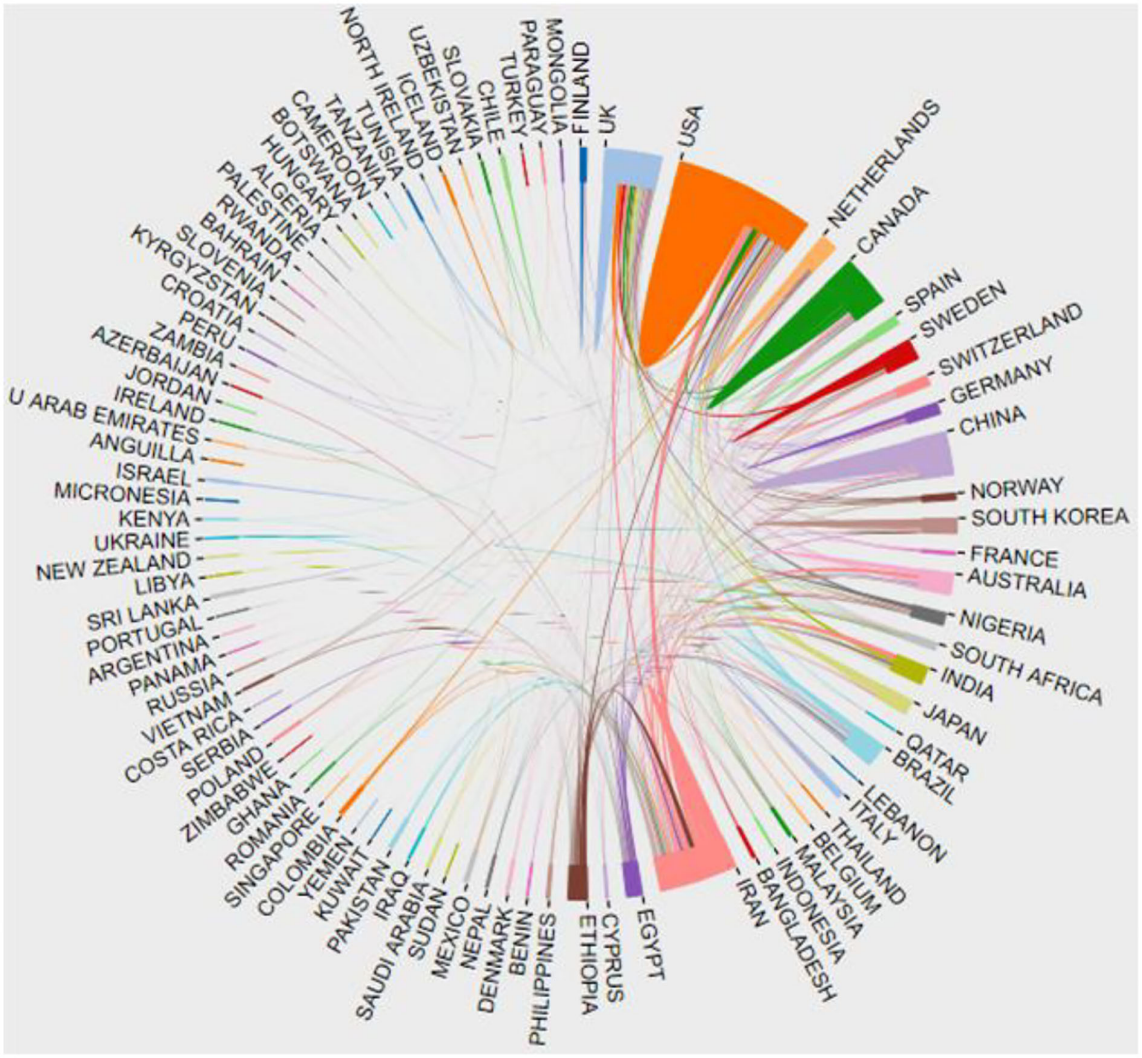
Cooperation between countries.

The cooperative relationship between countries is shown in Figure 4. Among them, authors from USA, UK, and IRAN had more cooperation with authors from other countries.

**Figure 5:**
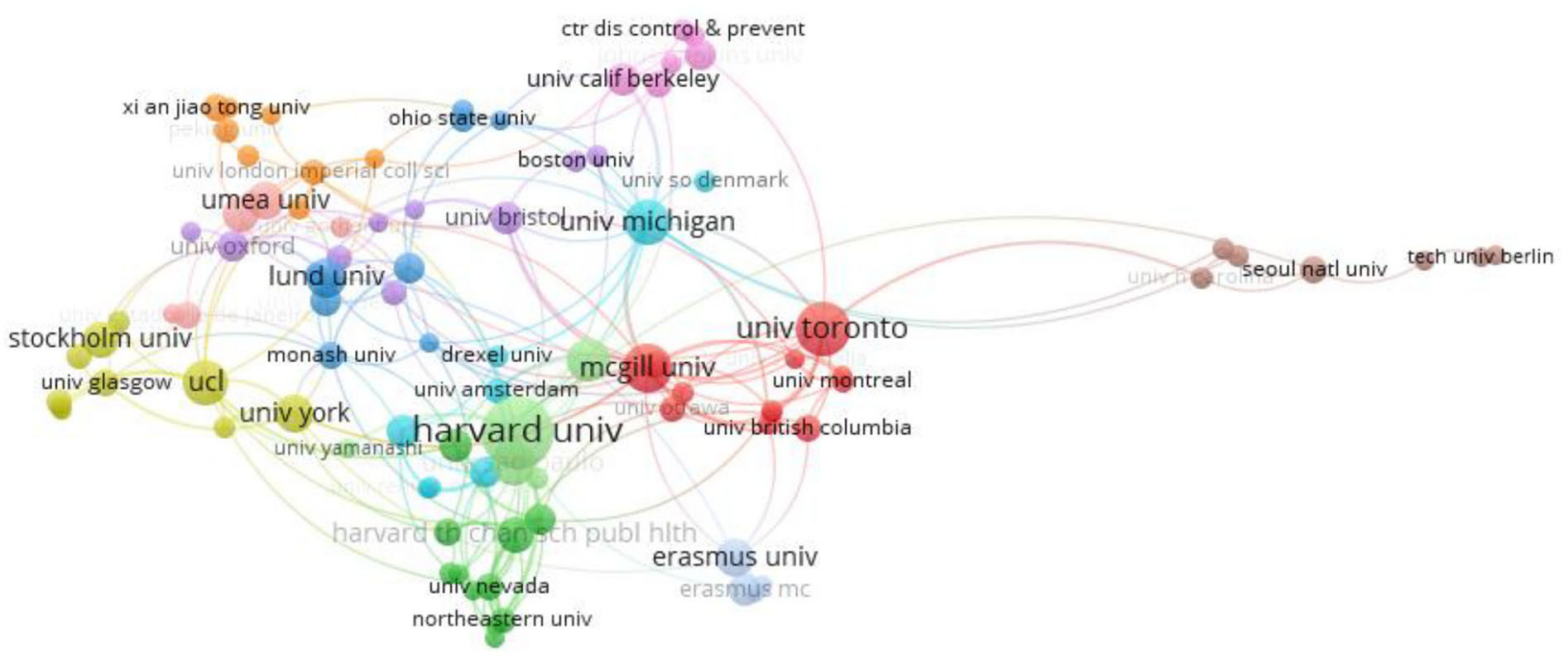
Cooperation between institution.

The cooperation between institutions is shown in Figure 5. Institutions include universities, research institutes, and national research institutions. The core of institutional cooperation network mainly included Harvard Univ, Univ Toronto, and Univ Michigan.

**Figure 6:**
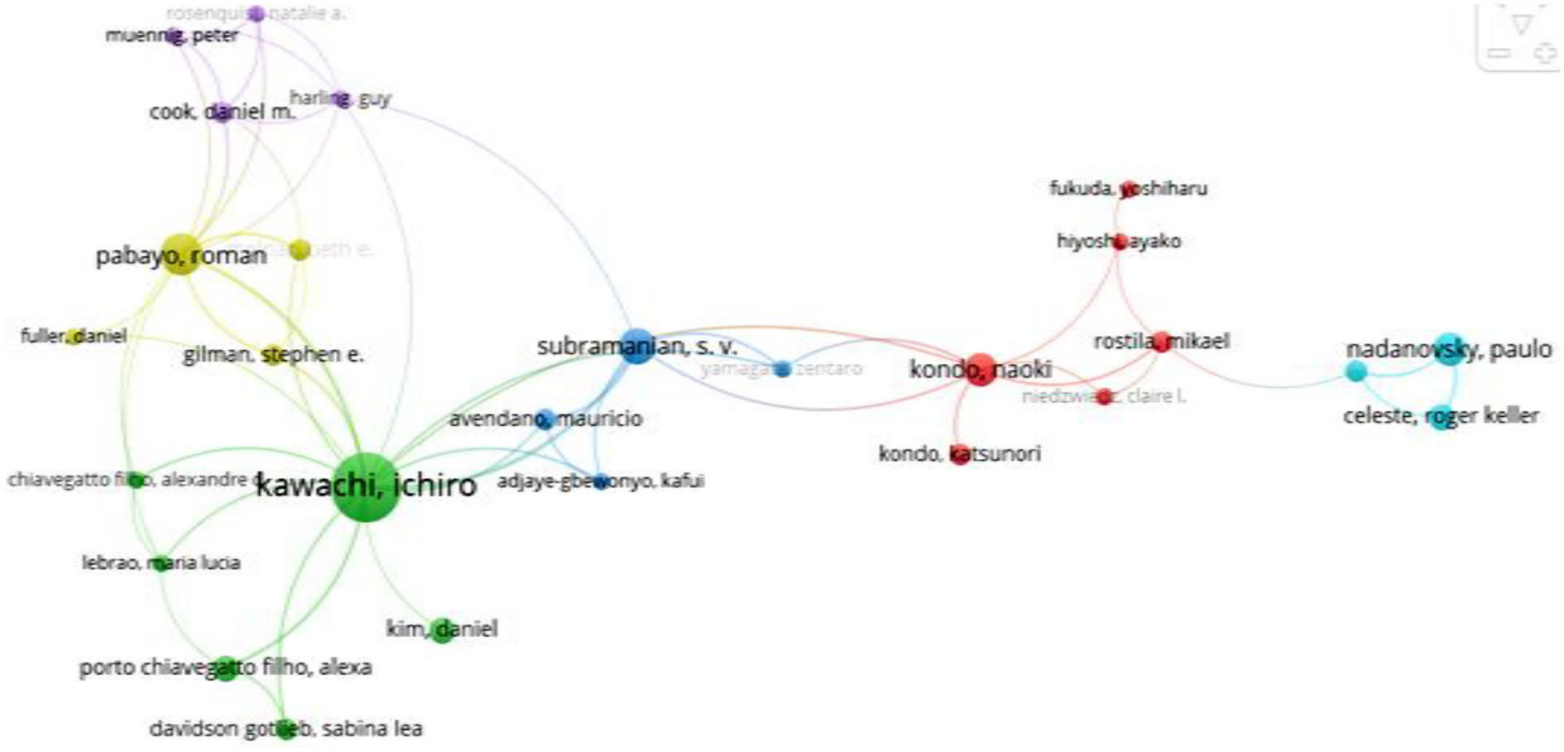
Collaboration between authors.

The cooperation between authors is shown in Figure 6. In the main author cooperation network, the authors who played a key role included Kawachi I, Subramanian SV, and Pabayo R.

### Influence analysis

The top 10 institutions with influence are shown in Table 2. Harvard Univ, Univ Michigan, and Natl Ctr Hlth Stat ranked the top three in the total number of references.

**Table 2:** Top 10 institutions with influence.

**Institution name**	**Total references**	**Total number of articles**	**Average cited times**	**Total number of first authors**	**Number of citations of the first author**	**Average citation of the first author**
Harvard Univ	872	65	13.42	22	601	27.32
Univ Michigan	211	21	10.05	2	61	30.50
Natl Ctr Hlth Stat	150	4	37.50	1	10	10
Columbia Univ	103	10	10.30	3	0	0
Erasmus Univ	101	15	6.73	8	77	9.63
Univ Notre Dame	100	4	25	2	50	25
Lund Univ	95	20	4.75	5	8	1.60
Univ Toronto	85	36	2.36	11	53	4.82
McGill Univ	85	19	4.47	7	28	4
Univ York	11	83	7.55	5	62	12.40

The top 10 publication titles with influence are shown in Table 3. “Social Science & Medicine,” “American Journal of Public Health,” and “Journal of Epidemiology and Community Health” ranked the top three in the total number of references.

**Table 3:** Top 10 publication titles with influence.

**Publication titles**	**Total references**	**Total number of articles**	**Average cited times**
Social Science & Medicine	679	100	6.79
American Journal of Public Health	308	22	14
Journal of Epidemiology and Community Health	288	46	6.26
Health Services Research	116	4	29
Epidemiologic Reviews	99	1	99
Health Economics	88	15	5.87
Journal of Health Economics	84	14	6
International Journal of Epidemiology	81	7	11.57
Health & Place	56	13	4.31
Milbank Quarterly	42	1	42

The top 10 authors with influence are shown in Table 4. Kawachi I, Kennedy BP, and Subramanian SV ranked the top three in the total number of references.

**Table 4:** Top 10 authors with influence.

**Author**	**Total references**	**Total number of articles**	**Average cited times**	**Total number of first authors**	**Number of citations of the first author**
Kawachi, I	662	37	17.89	3	222
Kennedy, BP	329	7	47	1	19
Subramanian, SV	223	13	17.15	5	189
Lochner, K	200	4	50	1	48
Kaplan, GA	125	5	25	0	0
Lynch, JW	96	2	48	1	54
ProthrowStith, D	94	1	94	0	0
Gerdtham, UG	91	9	10.11	1	7
Blakely, TA	83	3	27.67	3	83
van Doorslaer, E	83	8	10.38	3	56

### Keyword co-occurrence analysis

**Figure 7:**
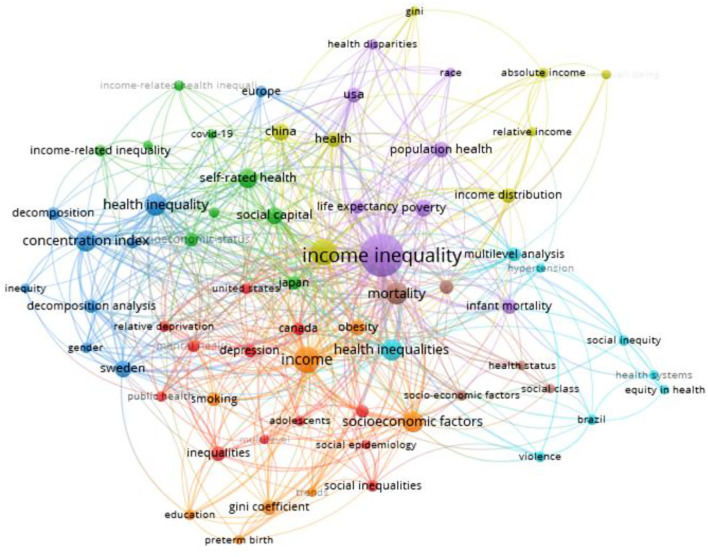
Keyword co-occurrence network.

The knowledge map of keyword co-occurrence can suggest hot topics (5). We made the keyword co-occurrence network according to author keywords from 1997 to 2021, as shown in Figure 7. Hot keywords included the income inequality, income, health inequality, mortality, socioeconomic factors, concentration index, social capital, self-rated health, income distribution, infant mortality, and population health.

### Co-citation analysis

The co-citation map of references estimated the scientific relevance of publications (18). Co-citation cluster analysis was used to explore the trends of the income inequality research in health field. Timeline visualization in CiteSpace described clusters through a horizontal timeline. Each cluster was displayed in left-to-right order. The largest cluster was shown at the top of the view. Large nodes or nodes with a red tree structure are particularly interesting because they are either highly referenced, have referenced bursts, or both (8). Representative publications are the documents with high co-citation frequency in each cluster, which influences the labeling of each cluster to reveal the research frontier. To better understand the development of the income inequality research in health field, we carried out co-citation analysis in three periods.

#### Timeline view (1997–2003)

**Figure 8:**
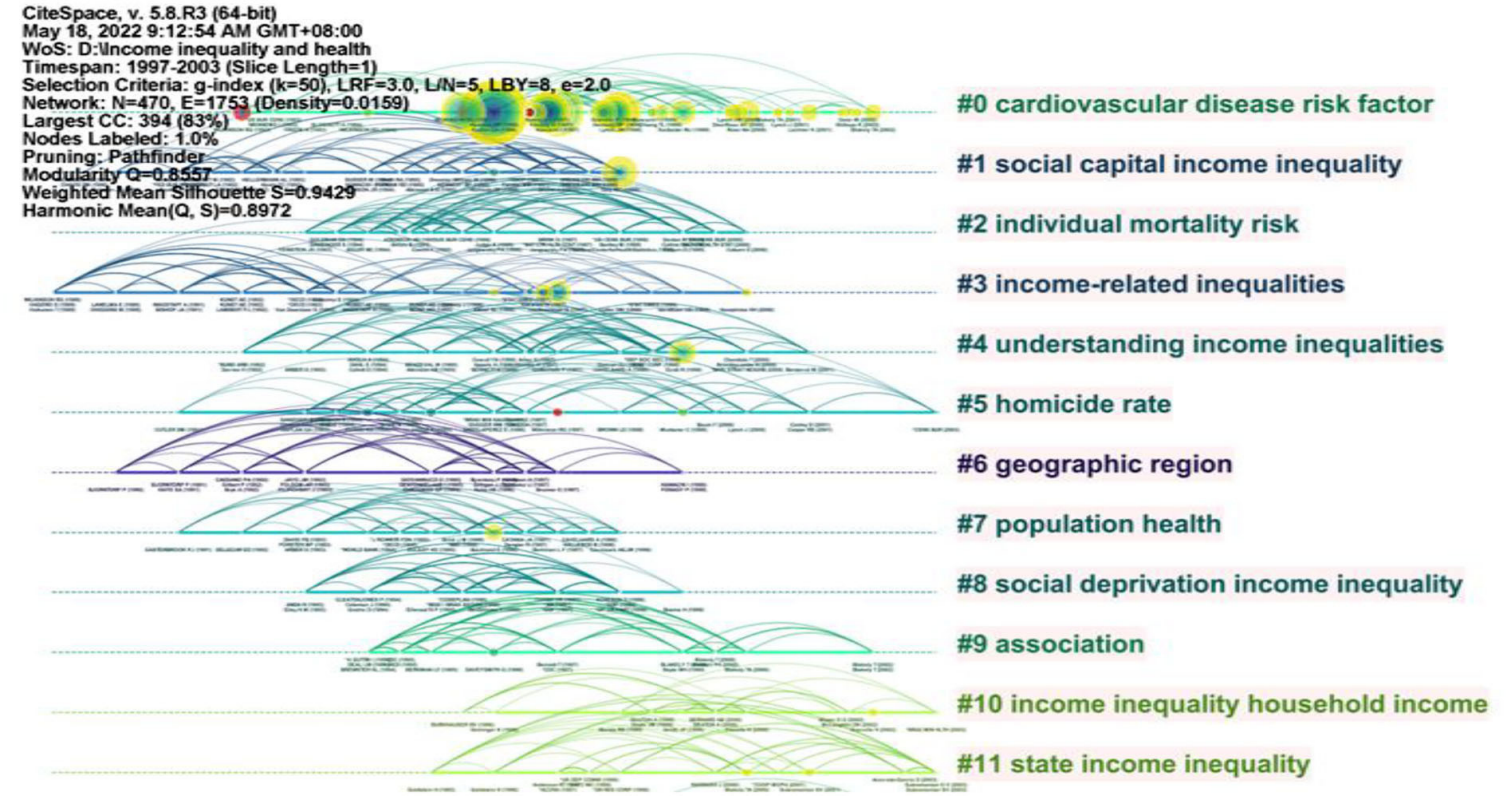
Timeline visualization of clusters in 1997–2003.

Based on the literature records from 1997 to 2003, we generated a cited reference map with 470 nodes and 1753 links (Figure 8). The results show the mean Silhouette (S = 0.9429) and the modularity (Q = 0.8557), and the modular Q value was greater than 0.7, which indicated that it was reasonable for the network to be divided into loosely coupled clusters (19). This paper did not analyze all clusters, but only some large clusters and clusters with newer mean year.

Cluster #0 was the largest cluster and represented “cardiovascular disease risk factor” with 45 members. In the representative publications, (20) used the data of behavioral risk factor monitoring system to test whether the uneven income distribution in the United States is related to four risk factors, such as body mass index (BMI), history of hypertension, sedentary disease, and smoking. The results about BMI, hypertension, and sedentary disease show that state inequality is associated with increased levels of risk factors, and this association persists while changing the individual income level. In addition, the correlation between inequality and smoking is positive. Moreover, this correlation is stronger than the other three factors (20).

Cluster #1 represented “social capital income inequality” with 44 members. In the representative publications, Kawachi et al. (2014) find that the widening gap between the rich and the poor has an impact on the social organization of the community. Therefore, the damage to the social structure may have a far-reaching impact on public health (21).

Cluster #2 represented “individual mortality risk” with 38 members. In the representative publications, Lochner et al. (2014) prove that income distribution has an important impact for health through adjusted income to find state-level income inequality has contextual effect on the risk of death. Meanwhile, the state income inequality will contextually affect personal death risk when controlling the national poverty level and individual sociodemographic characteristics (22).

Cluster #3 represented “income-related inequalities” with 37 members. In the representative publications (23), use the evidence of income and inequalities in self-assessed health for nine countries to suggest that inequalities in income are significantly correlated with inequalities in health (23). Gravelle and Sutton (2003) find that reducing income inequality and reducing the effect of income on health are helpful for reducing health inequality in pro-rich (24).

Cluster #4 represented “understanding income inequalities” with 29 members. In the representative publications (25), suggest that there is no threshold between health and income due to people in poverty which cannot represent overall situation. Furthermore, the household equivalent income should be taken as the key measure of income and adjusted according to the employment situation while deeply researching the relationship between income and health. In addition, men and women should be studied separately (25).

Cluster #10 represented “income inequality household income” with 18 members. In the representative publications (26), state that income inequality has no association with health situation. However, poor health is significantly correlated with low household income. It means that there is household income but not income inequality can explain some differences of health situation in Canada (26).

Cluster #11 represented “state income inequality” with 16 members. In the representative publications (27), used multilevel statistical models to detect the relationship between low self-assess of health and state income inequality. The result of their research shows that the difference of race has no effect on the association of income inequality and poor health situation in the United State (27).

Table 5 lists the detailed information of the references with strongest citation bursts in 1997–2003. The Sigma metric measures both citation burstness and structural centrality of a cited reference. Among them, (28) pointed out that mortality in developed countries was more affected by relative living standards than absolute living standards (28, 29) demonstrated that in most age groups, more equal income distribution was associated with lower all-cause mortality, which was contributed by all six major causes of death (29, 30) demonstrated that the association between income inequality and mortality observed in the United States at the state level cannot be fully or substantially explained as a statistical illusion of the potential individual-level relationship between income and mortality (30).

**Table 5:** References with strongest citation bursts in 1997–2003.

**Title**	**Strength**	**Begin**	**End**	**1997 - 2003**
Income distribution and life expectancy (Wilkinson, 1992; DOI 10.1136/bmj.304.6820.165)	3.90	1997	1999	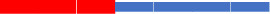
Income distribution and cause-specific mortality (Mcisaac & Wilkinson, 1997; DOI 10.1093/eurpub/7.1.45)	2.13	1999	2001	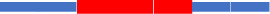
Socioeconomic determinants of health (Wilkinson, 1997; DOI 10.1136/bmj.314.7080.591)	3.62	2000	2001	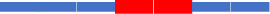
Relation between income inequality and mortality: empirical demonstration (Wolfson, 1999; DOI 10.1136/bmj.319.7215.953)	2.42	2001	2003	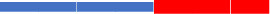

#### Timeline view (2004–2011)

**Figure 9:**
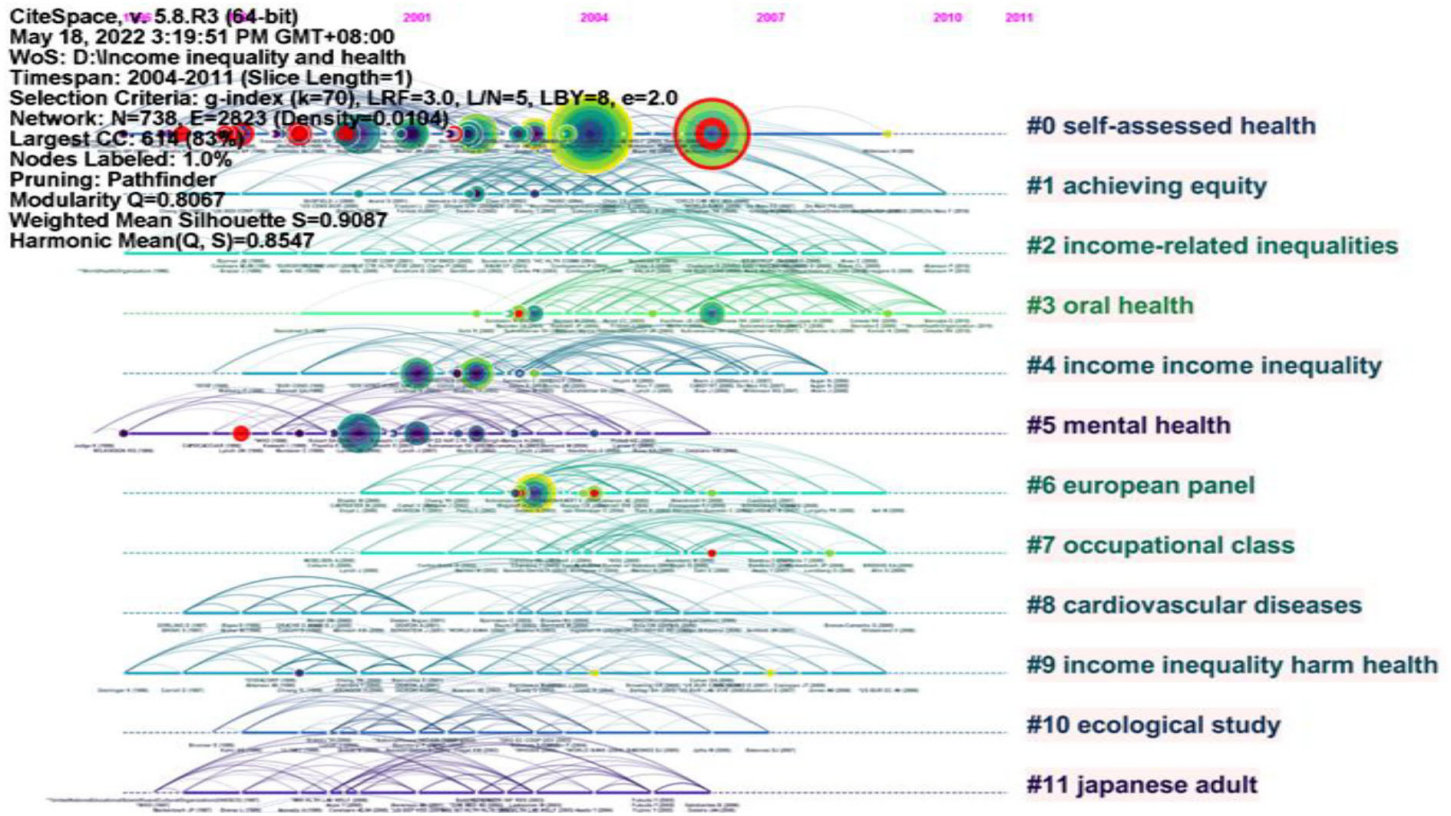
Timeline visualization of clusters in 2004–2011.

Based on the literature records from 2004 to 2011, we generated a cited reference map with 738 nodes and 2823 links (Figure 9). The results show the mean Silhouette (S = 0.9087) and the modularity (Q = 0.8067). This paper did not analyze all clusters, but only some large clusters and clusters with newer mean year.

Cluster #0 was the largest cluster and represented “self-assessed health” with 73 members. In the representative publications (31), finds that income inequality in Scotland is significantly positively correlated with health through single-level and multilevel logistic regression models. Moreover, the study also claims that the choice of methods to estimate the relationship between self-assessment health, individual socioeconomic status, and regional income inequality may not have a substantive impact on the result when the environmental impact is light (31). The research of (32) using the Gini coefficient, the Atkinson Index, and the Theil entropy index to analyze the association between income inequality and self-assess health finds that income inequality is significantly correlated with individual self-assess health (32). According to (33), the commonly used indicators of social capital and income inequality have a strong component impact on self-assess health (33).

Cluster #1 represented “achieving equity” with 52 members. In the representative publications (34), believe that some countries can overcome the adverse effects of income inequality on health by involving other health and social policies (34).

Cluster #2 represented “income-related inequalities” with 51 members. In the representative publications (35), measure inequality in England by the corrected concentration index and use instrumental variables regression to examine the endogeneity of income inequality and smoking. The results indicate that although the smoking rate has decreased, the contribution of smoking on income inequality has increased slightly over time because smoking is increasingly concentrated in the poor and has a negative impact on health (35, 36) find that population aging may affect income-related health inequality by age and gender (36).

Cluster #3 represented “oral health” with 51 members. In the representative publications (37), use the oral health data in Brazil and compare with individual-level covariates in regression. According to the result of the study, the effect of income inequality is mainly explained by public policy, and the independent effect of public policy is greater among the rich (37).

Cluster #5 represented “mental health” with 46 members. In the representative publications, Zimmerman and Bell (2006) according to the outcomes of self-reported general mental health status examine the relationship between individual health outcomes and ecological variables proposed in the causal relationship model between income inequality and health. In their findings, the impact of income inequality on health may be through the impact of objectionable social comparison and the reduction of social capital (38).

Cluster #6 represented “European panel” with 44 members. In the representative publications (39), used the panel data of the European community family group to detect the association between income and health. The results show that health and income growth can be coordinated only under very specific conditions on the type of growth and the income responsiveness of health (39).

Cluster #7 represented “occupational class” with 42 members. In the representative publications (4), find that people's health has improved significantly in all countries and people who have higher income than average lever is less likely to suffer from long-term diseases through used logistic regression to analyze two subjective health indicators (40).

Cluster #8 represented “cardiovascular diseases” with 36 members. In the representative publications (41), analyzed the cross-border relationship between income inequality and age-standardized mean BMI, serum total cholesterol, systolic blood pressure, obesity prevalence, smoking impact ratio, age-standardized and age-specific disability-adjusted life years, coronary heart disease, and moderate risk mortality and found that the higher the degree of income inequality, the higher the average systolic blood pressure of men and women, and the higher the smoking impact ratio of women (41).

Table 6 lists the detailed information of the top 10 references with strongest citation bursts in 2004–2011 (There were 13 references with the strongest citation bursts in 2004–2011, and we chose 10 representative references). The Sigma metric measures both citation burstness and structural centrality of a cited reference. Most references mainly studied the relationship between income inequality and mortality and the relationship between income inequality and population health. Among them, (42) found that the lack of high school education accounted for the income inequality effect and was a powerful predictor of mortality variation among United States (42, 43) found that relatively equal income distribution and generous and comprehensive welfare system did not hinder the impact of income inequality on mortality in Norway at the regional level, which was particularly significant among socioeconomic vulnerable groups (43), critically reviewed the published literature on the relationship between income inequality and health outcomes and found that at least 33 studies showed a significant association, while at least 12 studies did not find such an association (10); based on the research results of 155 reported on the relationship between income distribution and population health (44), made it clear that income inequality could be used as a measure of the scale of social stratification or social rank (44).

**Table 6:** Top 10 references with strongest citation bursts in 2004–2011.

**Title**	**Strength**	**Begin**	**End**	**2004–2011**
How much of the relation between population mortality and unequal distribution of income is a statistical artifact? (Gravelle, 1998; DOI 10.1136/bmj.316.7128.382)	4.36	2004	2005	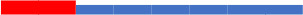
The relationship of income inequality to mortality: Does the choice of indicator matter? (Kawachi, 1997; DOI 10.1016/S0277-9536(97)00044-0)	3.99	2004	2005	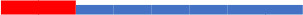
Education, income inequality, and mortality: a multiple regression analysis (Muller, 2002; DOI 10.1136/bmj.324.7328.23)	2.53	2004	2005	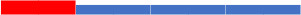
Relation between income inequality and mortality: empirical demonstration (Wolfson, 1999; DOI 10.1136/bmj.319.7215.953)	2.5	2004	2006	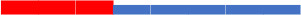
Income inequality and population health: A review and explanation of the evidence (Wilkinson, 2006; DOI 10.1016/j.socscimed.2005.08.036)	5.32	2008	2011	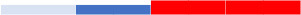
Explaining the differences in income-related health inequalities across European countries (van Doorslaer, 2004; DOI 10.1002/hec.918)	3.14	2009	2011	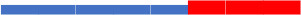
Income inequality and health: A critical review of the literature (Macinko, 2003; DOI 10.1177/1077558703257169)	2.94	2009	2011	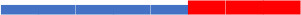
Income inequality and ischaemic heart disease in Danish men and women (Osler, 2003; DOI 10.1093/ije/dyg074)	2.24	2009	2011	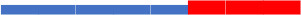
For whom is income inequality most harmful? A multi-level analysis of income inequality and mortality in Norway (Dahl, 2006; DOI 10.1016/j.socscimed.2006.06.002)	2.24	2009	2011	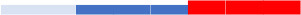
Income distribution, public services expenditures, and all cause mortality in US states (Dunn, 2005; DOI 10.1136/jech.2004.030361)	2.24	2009	2011	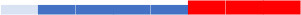

#### Timeline view (2012–2021)

**Figure 10:**
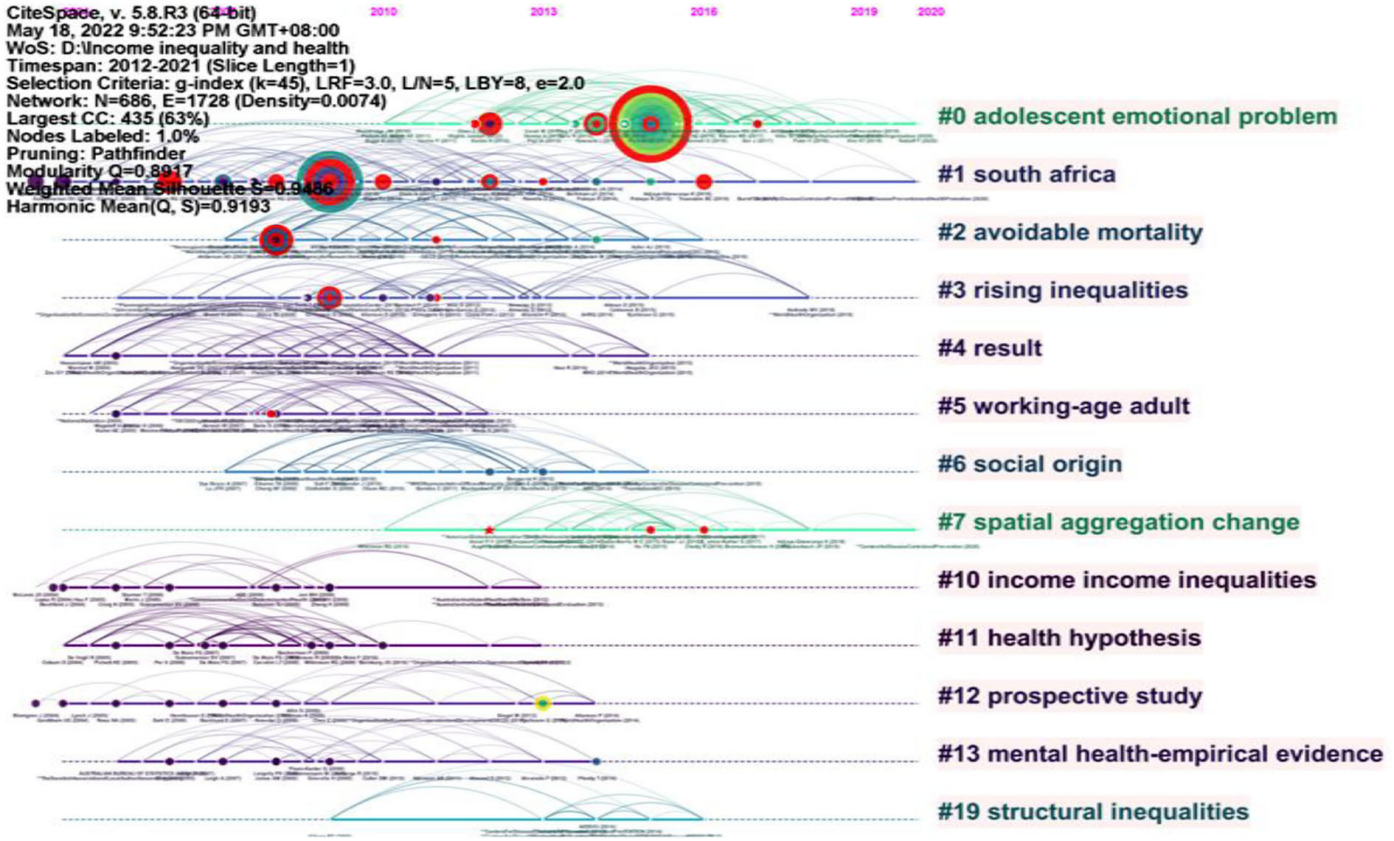
Timeline visualization of clusters in 2012–2021.

Based on the literature records from 2012 to 2021, we generated a cited reference map with 686 nodes and 1728 links (Figure 10). The results show the mean Silhouette (S = 0.9486) and the modularity (Q = 0.8971). This paper did not analyze all clusters, but only some large clusters and clusters with newer mean year.

Cluster #0 was the largest cluster and represented “adolescent emotional problem” with 70 members. In the representative publications (45), analyze the relationship between community income inequality and adolescent emotional problems and discuss whether these relationships depend on national income inequality and individual deprivation. The results show that the harmful link between income inequality and adolescent emotional problems may be determined by the degree of income inequality in the larger social context (45).

Cluster #1 represented “South Africa” with 65 members. In the representative publications (45, 46), investigate whether income inequality in South African countries is associated with cardiovascular disease, BMI, waist circumference, blood pressure, inactivity, smoking, and high alcohol consumption. The outcomes indicate that there is no relationship between income inequality and cardiovascular disease risk factors in South Africa (46, 47).

Cluster #2 represented “avoidable mortality” with 44 members. In the representative publications (48), use data from the Norwegian income register and the causes of death register, a register-based demographic study was conducted on Norwegian residents aged 18–65 between 1994 and 2011. The results show that income-related inequality in avoidable, compensable, and preventable mortality is mainly related to the relationship between income and avoidable mortality, rather than changes in the Gini coefficient of income inequality (48).

Cluster #3 represented “Rising inequalities” with 40 members. In the representative publications [50, examined the extent to which changes in income levels and distribution and income mobility are related to the health gap between the rich and the poor. This study points out that poor health is associated with income experience, but not with average income growth over the past few decades (49).

Cluster #4 represented “results from world health survey” with 31 members. In the representative publications (50, 51), used the world health survey data of 41 low-income countries from 2002 to 2004 and found that non communicable diseases show an unequal distribution in the socioeconomic groups of low-income countries (50, 51).

Cluster #5 represented “working-age adult” with 31 members. In the representative publications (52) state that income-related inequalities in sexual and reproductive health persisted to the detriment of low-income groups between 1986 and 2007 after analyzing a nationally representative sample of Japanese men and women aged 20–59 (52).

Cluster #7 represented “spatial aggregation change” with 27 members. In the representative publications (53), used a large, nationally representative elderly data set to detect the relationship between income inequality (Gini index) and population health. The results show that the association between income inequality and health varies with the level of spatial aggregation (53).

Cluster #12 represented “prospective study” with 18 members. In the representative publications (54), calculated the HRS and 95% CI of income inequality and post-working-age mortality in 140 urban areas based on immigration status, personal and neighborhood income, and sociodemographic characteristics. According to their findings, the correlation between income inequality and mortality among individuals born is positive in Canada. Meanwhile, there is no association between income inequality and immigration (54).

Cluster #13 represented “mental health-empirical evidence” with 17 members. In the representative publications (55), analyze the association between mental health and inequality in Australia and examine the hypothesis of income inequality and relative deprivation by calculating various income inequality indexes. The results indicate that mental health will only be negatively affected by relative deprivation to a light level (55).

Table 7 lists the detailed information of the top 17 references with strongest citation bursts in 2004–2011 (There were 21 references with the strongest citation bursts in 2012–2021, and we chose 17 representative references). The Sigma metric measures both citation burstness and structural centrality of a cited reference. Among them (56), found that income inequality has a certain degree of adverse impact on health through meta-analysis, the results also support the threshold effect hypothesis, which assumes that there is a threshold of income inequality, and the adverse impact on health beyond this threshold begins to appear (56, 57) investigated whether the processes associated with income inequality and population health are related to those responsible for the socioeconomic gradient of health and whether the health difference is small when the income difference is small. It was found that the impact of state income inequality on the socioeconomic gradient of health varies according to the cause of death, but greater equality usually benefited both wealthier and poorer counties (57, 58) assessed the association between income inequality and life expectancy in Brazil between 2000 and 2009, including the impact of social and health interventions. It found that Gini index, as another measure of income inequality, was negatively correlated with life expectancy. The country's main primary healthcare (PHC) program, family health program, was positively correlated with life expectancy (58, 59) pointed out that low-income Americans are increasingly being left behind because they have missed decades of income growth and longevity growth. If no intervention measures are taken to decouple income from health or reduce income inequality, the health poverty trap will appear in the 21st century, and the socioeconomic inequality in health will further expand and intensify (59, 60) used comparable data between 1987 and 2008 to critically assess the relationship between income inequality and mortality in 43 European countries. After controlling for time invariant and time-varying national confounding factors, it was found that there was a significant correlation between income inequality and many mortality indicators (60).

**Table 7:** Top 17 references with strongest citation bursts in 2012–2021.

**Title**	**Strength**	**Begin**	**End**	**2012–2021**
Income inequality and population health: A review and explanation of the evidence (Wilkinson, 2006; DOI 10.1016/j.socscimed.2005.08.036)	6.78	2012	2014	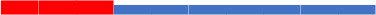
Income inequality, mortality, and self rated health: meta-analysis of multilevel studies (Kondo, 2009; DOI 10.1136/bmj.b4471)	3.59	2012	2015	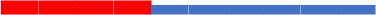
Correcting the Concentration Index (Erreygers, 2009; DOI 10.1016/j.jhealeco.2008.02.003)	5.42	2013	2015	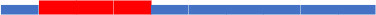
Mortality, lifestyle and socio-economic status (Balia, 2008; DOI 10.1016/j.jhealeco.2007.03.001)	2.72	2013	2014	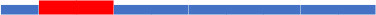
Measuring socioeconomic inequality in health, health care and health financing by means of rank-dependent indices: A recipe for good practice (Erreygers, 2011; DOI 10.1016/j.jhealeco.2011.04.004)	2.72	2013	2014	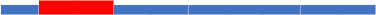
Income inequality and socioeconomic gradients in mortality (Wilkinson, 2008; DOI 10.2105/AJPH.2007.109637)	2.65	2013	2016	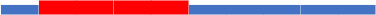
Socioeconomic inequalities in health in 22 European countries (Mackenbach, 2008; DOI 10.1056/NEJMsa0707519)	3.22	2014	2016	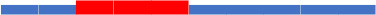
Do people die from income inequality of a decade ago? (Zheng, 2012; DOI 10.1016/j.socscimed.2012.02.042)	2.97	2015	2018	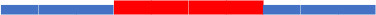
Income inequality, trust, and population health in 33 countries (Elgar, 2010; DOI 10.2105/AJPH.2009.189134)	2.76	2015	2016	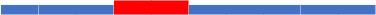
Income inequality and health: A causal review (Pickett, 2015; DOI 10.1016/j.socscimed.2014.12.031)	5.38	2016	2021	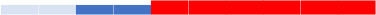
Impact of income inequality on life expectancy in a highly unequal developing country: The case of Brazil (Rasella, 2013; DOI 10.1136/jech-2012-201426)	2.56	2017	2018	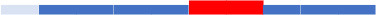
The health effects of income inequality: Averages and disparities (Truesdale, 2016; DOI 10.1146/annurev-publhealth-032315-021606)	3.06	2018	2019	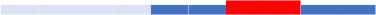
Population health in an era of rising income inequality: USA, 1980–2015 (Bor, 2017; DOI 10.1016/S0140-6736(17)30571-8)	2.75	2018	2021	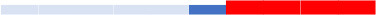
Income inequality, life expectancy and cause-specific mortality in 43 European countries, 1987–2008: a fixed effects study (Hu, 2015; DOI 10.1007/s10654-015-0066-x)	2.55	2018	2019	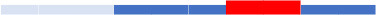
Income inequality and health: the role of population size, inequality threshold, period effects and lag effects (Kondo, 2012; DOI 10.1136/jech-2011-200321)	2.51	2018	2019	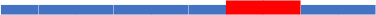
The association between income and life expectancy in the United States, 2001-2014 (Chetty, 2016; DOI 10.1001/jama.2016.4226)	2.92	2019	2021	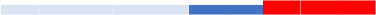
The role of geographic scale in testing the income inequality hypothesis as an explanation of health disparities (Chen, 2012; DOI 10.1016/j.socscimed.2012.04.032)	2.75	2019	2021	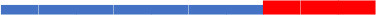

### Structural variation analysis (SVA)

**Figure 11:**
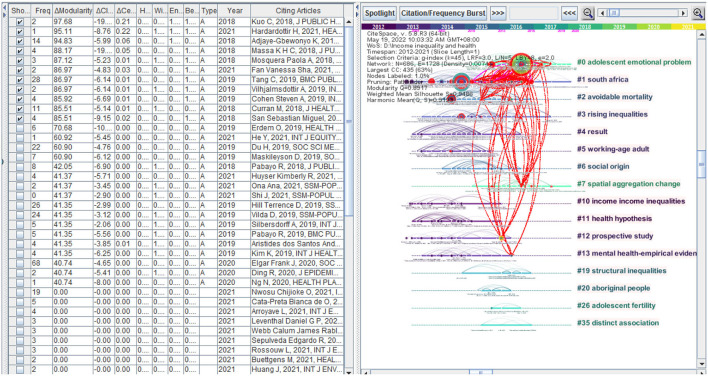
Timeline visualization of clusters and SVA's result in 2012–2021.

The major limitation of any citation-based indicators is their reliance on citations accumulated over time. Thus, citation-based indicators are likely to overlook newly published articles. An alternative method (SVA) is to focus on the extent to which a newly published article brings to the conceptual structure of the knowledge domain of interest (61). The SVA procedure looks for newly added connections that may alter the global structure or have the potential to do so (62). The idea is to identify the potential of an article to make extraordinary or unexpected connections across distinct clusters. According to theories of scientific discovery, many significant contributions resulted from boundary-spanning ideas (8). To measure the transformative potentials of recent papers, we use the SVA of CiteSpace (We used 6-year span sliding windows). Figure 11 shows SVA's result in 2012–2021. The red line on the right side of the figure indicates that the papers with a high transformative potential span different clusters, reflecting a certain degree of intersection.

Table 8 shows a list of articles with high transformative potentials based on the modularity. There are 6 papers in 2018, three papers in 2019, and 2 papers in 2021. Among them, Kuo and Chen (2018) found that the joint effects of income inequality and polarization of small-scale economies might reveal how inequality increases mortality (63, 64) found that the income inequality had a 139.7 to 374.3% more harmful effect on health in poorer than richer countries, and a significantly harmful effect in 2.1 to 53.3% of countries in their sample and 6.6 to 67.6% of the world's population, but no significantly harmful effect in richer countries (64, 65) used an Internet-based survey to ask whether a representative sample of the Swedish population supports the ethical assumptions of the concentration index of income-related health inequality and the Gini index of income inequality and found that the median subjects' preference for income-related health inequality was consistent with the ethical hypothesis implied by the concentration index, but the weight for the poor was higher than that implied by the Gini index of income inequality (65).

**Table 8:** Some of the articles with the strongest transformative potentials, M(ΔModularity), C-L(ΔCluster Linkage), and C-D(ΔCentrality Divergence).

**Year**	**M**	**C–L**	**C–D**	**Title**	**References**
2018	97.68	−19.4	0.21	Double disadvantage: income inequality, spatial polarization and mortality rates in Taiwan	Kuo C. 2018, J Public Health–UK, V40, PE228, DOI 10.1093/pubmed/fdx179
2021	95.11	−8.76	0.22	Parameterizing standard measures of income and health inequality using choice experiments	Hardardottir H, 2021, health Econ, V30, P2531, DOI 10.1002/hec.4395
2018	94.83	−5.99	0.06	Income inequality and cardiovascular disease risk factors in a highly unequal country: A fixed–effects analysis from South Africa	Adjaye–Gbewonyo K, 2018, int J Equity Health, V17, P0, DOI 10.1186/s12939–018–0741–0
2018	88.17	−19.79	0.05	Income inequality and self–reported health in a representative sample of 27 017 residents of state capitals of Brazil	Massa K H C, 2018, J public Health–UK, V40, PE440, DOI 10.1093/pubmed/fdy022
2018	87.12	−5.23	0.01	Decomposition of gendered income–related inequalities in multiple biological cardiovascular risk factors in a middle–aged population	Mosquera Paola A, 2018, Int J Equity Health, V17, P0, DOI 10.1186/s12939–018–0804–2
2019	86.97	−6.14	0.01	Examining income–related inequality in health literacy and health–information seeking among urban population in China	Tang C, 2019, BMC Public Health, V19, P92, DOI 10.1186/s12889–019–6538–2
2019	86.97	−6.14	0.01	Decreasing income inequality and adolescent emotional distress: A population–based case study of Icelandic adolescents 2006–2016	Vilhjalmsdottir A, 2019, Int J Public Health, V64, P253, DOI 10.1007/s00038–018–1193–4
2021	86.97	−4.83	0.03	Effect of income inequality, community infrastructure and individual stressors on adult depression	Fan Vanessa Sha, 2021, Health Promot Int, V36, P46, DOI 10.1093/heapro/daaa036
2019	85.92	−6.69	0.01	A “Swiss paradox” in the United States? Level of spatial aggregation changes the association between income inequality and morbidity for older Americans	Cohen Steven A, 2019, Int J Health Geogr, V18, P0, DOI 10.1186/s12942–019–0192–x
2018	85.51	−5.14	0.01	Income inequality and population health: A global gradient?	Curran M, 2018, J Health Soc Behav, V59, P536, DOI 10.1177/0022146518808028
2018	85.51	−9.15	0.02	Whose income is more important: mine, yours or ours? Income inequality and mental health in northern Sweden	San Sebastian Miguel, 2018, Eur J Public Health, V28, P1056, DOI 10.1093/eurpub/cky110

## Conclusion and discussion

The use of interactive visualization considerably reduces the cognitive load of visual exploration of scientific literature. This study utilizes a bibliometric approach to review the discipline co-occurrence, publication characteristics, partnership, keyword co-occurrence, co-citation, research themes, and the transformative potentials of recent papers within the income inequality research in health field. We advance the following main conclusions:

First, we found that more source journals are located in “2. MEDICINE, MEDICAL, CLINICAL,” “6. PSYCHOLOGY, EDUCATION, HEALTH,” and “9. DENTISTRY, DERMATOLOGY, SURGERY.”

Second, we found that the USA contributed most articles, the Harvard Univ was the most influential institution, Social Science & Medicine was the most influential journal, and Kawachi I was the most influential author.

Third, we found that the main hotspots included the income inequality, income, health inequality, mortality, socioeconomic factors, concentration index, social capital, self-rated health, income distribution, infant mortality, and population health in 1997–2021.

Fourth, we found that the cardiovascular disease risk factor, social capital income inequality, individual mortality risk, income-related inequalities, understanding income inequalities, income inequality household income, and state income inequality had been the hot research topics in 1997–2003; the self-assessed health, achieving equity, income-related inequalities, oral health, mental health, European panel, occupational class, and cardiovascular diseases had been the hot research topics in 2004–2011; the adolescent emotional problem, South Africa, avoidable mortality, rising inequalities, results from world health survey, working-age adult, spatial aggregation change, prospective study, and mental health-empirical evidence had been the hot research topics in 2012–2021.

Last, we revealed that there were 11 articles with strong transformation potential during 2012–2021, six papers in 2018, three papers in 2019, and two papers in 2021.

The limitations of this study are shown as follows:

On the one hand, the data only come from the English papers in WoS Core Collection, resulting in the exclusion of a large number of English documents in Scopus, PubMed/Medline, and other databases, as well as a large number of documents in other language countries (66). It is necessary to conduct bibliometric analysis of the related literature in other languages.

On the other hand, due to the limitation of the CiteSpace software itself, some important research topics in the relationship of the income inequality and population health might be omitted using CiteSpace for co-citation analysis, for example, the relationship between COVID-19 and income inequality (67–70). This important research topic is worthy of further bibliometric analysis in future.

## Data availability statement

The raw data supporting the conclusions of this article will be made available by the authors, without undue reservation.

## Author contributions

GX and JL: conceptualization. MD: methodology and data curation. JL: formal analysis. JL, SZ, and MD: writing—original draft preparation. JL and SZ: writing—review and editing. GX: funding acquisition. All authors have read and agreed to the published version of the manuscript.

## Funding

This study was supported by the National Social Science Foundation of China (20BTJ011) and the Guangxi Natural Science Foundation (2021GXNSFBA196044).

## Conflict of interest

The authors declare that the research was conducted in the absence of any commercial or financial relationships that could be construed as a potential conflict of interest.

## Publisher's note

All claims expressed in this article are solely those of the authors and do not necessarily represent those of their affiliated organizations, or those of the publisher, the editors and the reviewers. Any product that may be evaluated in this article, or claim that may be made by its manufacturer, is not guaranteed or endorsed by the publisher.
